# The Influence of the Strain Rate and Prestatic Stress on the Dynamic Mechanical Properties of Sandstone—A Case Study from China

**DOI:** 10.3390/ma16093591

**Published:** 2023-05-08

**Authors:** Jun Wang, Zhiwei Ren, Shang Yang, Jianguo Ning, Shuai Zhang, Yongtian Bian

**Affiliations:** 1 State Key Laboratory of Water Resource Protection and Utilization in Coal Mining, China Energy Investment Group, Beijing 102209, China; 2 State Key Laboratory of Mining Disaster Prevention and Control Co-Founded by Shandong Province and the Ministry of Science and Technology, Shandong University of Science and Technology, Qingdao 266590, China; 3 State Key Laboratory of Mining Response and Disaster Prevention and Control in Deep Coal Mine, Anhui University of Science and Technology, Huainan 232001, China

**Keywords:** SHPB, dynamic strength, strain rate, prestatic stress, microfracturing, failure mechanisms

## Abstract

A series of conventional dynamic uniaxial compressive (CDUC) tests and coupled static dynamic loading (CSDL) tests were performed using a split Hopkinson compression bar (SHPB) system to explore the variable dynamic mechanical behavior and fracture characteristics of medium siltstone at a microscopic scale in the laboratory. In the CDUC tests, the dynamic uniaxial strength of the medium sandstone is rate-dependent in the range of 17.5 to 96.8 s^−1^, while the dynamic elastic modulus is not dependent on the strain rate. Then, this paper proposes a generalized model to characterize the rate-dependent strength from 17.5 to 96.8 s^−1^. In the CSDL tests, with increasing initial prestatic stress, the dynamic elastic modulus and dynamic strength increase nonlinearly at first and then decrease. The results show that two classical morphological types (i.e., Type I and Type II) are observed in the dynamic stress–strain response from the CDUC and CSDL tests. By scanning electron microscopy (SEM), microscopic differences in the post-loading microcrack characteristics in the behavior of Type I and Type II are identified. In Class I behavior, intergranular fracture (IF) usually initiates at or near the grains, with most cracks deflected along the grain boundaries, resulting in a sharp angular edge, and then coalesces to the main fracture surface that splits the specimen along the direction of stress wave propagation. In contrast, Class II behavior results from the combined IF and transgranular fracture (TF).

## 1. Introduction

Rocks can be exposed to high loading rates and prestatic stress in many engineering fields, such as tunneling, exploration drilling for oil, gas, mining and blasting applications [1,2,3,4]. Substantial studies on the effects of strain rates and prestatic stress on rock mechanical properties and macrobreakage behavior have been performed in recent years [5,6,7,8,9]. Generally, the onset of microbreakage behaviors undergoes the process of microcrack initiation, propagation and coalescence. While the trajectory of the macrofracture exhibits a pronounced strain rate and prestatic stress sensitivity, the effects of the strain rate and pre-stress on microscopic damage need to be investigated.

Some researchers have performed experimental studies and numerical simulations on the microfractures of grain-based geomaterials that deviate from the behavior for quasistatic conditions. Microexperimental examinations showed that failure under quasistatic loading usually occurs along the interface of cemented geomaterials, whereas, with an increase in the loading rate, cracks penetrate the grains directly [10,11,12,13,14]. For grain-based geomaterials, two main types of macroscopic failure patterns occur: intergranular fractures and transgranular fractures [15,16,17]. In terms of energy, this micro-difference in the failure pattern is clarified by the theory of fracture mechanics. However, understanding the effects of the loading rate on the microscopic failure pattern is sometimes challenging.

In recent years, the dynamic mechanical properties of rock have attracted wide concern. Previous studies have shown that rock mechanical properties depend on the strain rate in terms of their stress–strain response as well as their strength [18,19,20]. In addition, some researchers have noted that the dynamic increase factor (DIF) shows a non-linear increase in the strain rate over the full range. This change includes a slight rate dependence in the range of 100–105 and a strong rate independence at ultrahigh strain rates [21]. Then, different empirical laws are presented to characterize the change at a given strain rate.

However, there is a poor ability to characterize intermediate strain-rate behavior. To study the dynamic fracturing of quartz monzonite samples, Aben et al. [22] performed laboratory tests using an SHPB and found three classical mechanical types with strain rates from 10 to 385 s^−1^. Mechanical Class I samples are mainly affected by inelastic deformation, and samples generally do not fail. Mechanical Class II samples undergo sufficient damage to fail when subjected to pervasive dynamic fracturing. Class III samples are qualitatively described as pulverized. Based on experimental results, Li et al. [23] noted that Class I failure mechanisms change from intact to single fractured or lightly fragmented, and the Class II failure mechanism is characterized by pulverized fragments.

These investigations showed that dynamic fracturing failed to reach an agreement. Generally, the onset of macrocracks and fractures undergoes the process of microcrack initiation, propagation and coalescence, which is widely encountered in natural grain-based geomaterials. This behavior suggests that understanding microscale failure may provide a way to agree on the differences in microfractures.

Aside from compressive dynamic loading, coupled static–dynamic loading is also widespread in mining and geotechnical engineering [24,25,26]. Generally, the different loading conditions result in different rock mechanical properties and failure behavior. Yao et al. [27] and Wu et al. [28] studied the influence of prestatic stress on dynamic mechanical properties and concentrated on micro-experimental investigations. However, an adequate theoretical study requires detailed consideration of the microscopic characteristics of fracture when studying the effect of prestress on the mechanical behavior of rocks. Microscopic differences in rock microfractures must, therefore, be investigated under different coupled static–dynamic loading conditions.

In this test, SHPB equipment is used to conduct dynamic impact tests on typical medium sandstone under different dynamic load conditions. To investigate the effects of the strain rate, dynamic loading experiments in compression are conducted on a series of medium sandstone samples. A second series of samples is subjected to coupled static–dynamic loading conditions. The incident wave reflected wave and transmitted wave are monitored. We perform an electron microscope scanning test on the sample after dynamic load damage to determine the microscopic morphology of the sample fracture.

## 2. Experimental Methodology

### 2.1. Specimen Preparation

The medium sandstone (sandstone particle size is 0.5–0.25 mm) in this test was obtained from Inner Mongolia Coal Mine in China and transported to the State Key Laboratory for Mine Disaster Prevention and Control of Shandong University of Science and Technology for processing. After drilling, cutting and polishing, it conformed to ISRM standards [29]. The size of the test sample was 50 × 50 mm standard cylindrical, the average elastic modulus of the sample was 6.73 GPa, the average uniaxial compressive strength was 76.6 MPa, and the average Poisson’s ratio was 0.28.

### 2.2. Experimental Apparatus Testing Program

**Figure 1:**
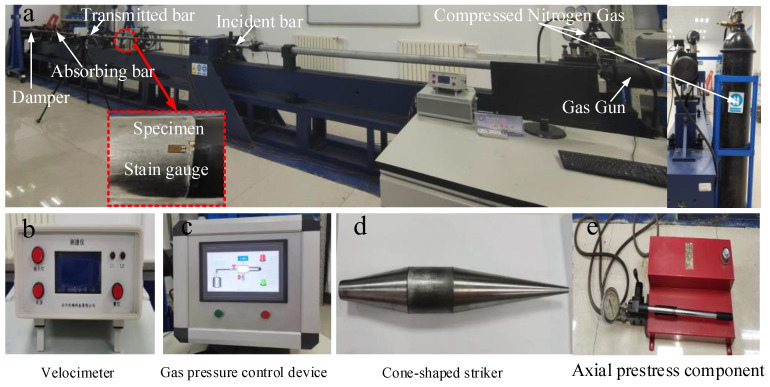
Diagram of the SHPB experiment system.

All laboratory tests were performed on a modified SHPB mounting system as indicated in Figure 1. The relevant parameters of the equipment have been introduced in published articles [30] and will not be detailed in this section. In the dynamic load test, the spindle bullet in the gas cylinder is driven by nitrogen to hit the incident rod, and the kinetic energy of the bullet is transmitted in the form of a wave in the compression rod.

Due to the different materials of the test piece, the reflection and transmission ability of the wave are different, so the change of the wave was monitored by placing strain gauges on the incident rod and transmission rod, and the strain data were obtained by processing the three-wave method provided in [31]. The axial stress of the sample can be obtained as σ(t) strain ε(t) and strain rate $\dot{\varepsilon }(t)$ along with other mechanical parameters. The specific calculation method is as follows.

(1) $$\sigma (t)=\frac{A_{0}E_{0}}{2A_{S}}[\sigma_{i}(t)+\sigma_{r}(t)+\sigma_{t}(t)]$$

(2) $$\varepsilon (t)=\frac{c_{0}}{l_{0}}\int_{0}^{t}\left[\sigma_{i}(t)-\sigma_{r}(t)-\sigma_{t}(t)\right]dt$$

(3) $$\dot{\varepsilon }(t)=\frac{c_{0}}{l_{0}}[\sigma_{i}(t)-\sigma_{r}(t)-\sigma_{t}(t)]$$

where *A*_0_, *E*_0_ and *C*_0_ are the cross-sectional area, Young’s modulus and P-wave velocity of the elastic bar, respectively. *A_s_* and *L*_0_ are the transverse area and length of the sample respectively.

Li et al. [32] demonstrated that one-dimensional elastic wave transmission is still valid for static–dynamic compression tests with axial coupling. This conclusion has been widely cited by some scholars. Thus, we used the three-wave analysis method for data processing.

The impact speed can be controlled by adjusting the nitrogen gas pressure. Through the speed calibration test, we determined that there was an obvious linear relationship between the impact speed and the gas pressure (Formula (4)).

(4) $$P_{s}=5.168P_{0}+4.033$$

where *P*_0_ is the gas pressure. *Ps* is the striker’s speed.

### 2.3. Testing Procedure

Zhou et al. [31] recommended that the axial prestress should not be more than 80% of the UCS because of the potential for self-sustaining damage to the specimen prior to impact. The UCS of this rock material was approximately 76.6 MPa, and the desired axial prestatic stress should not have exceeded 61.28 MPa.

Thus, this dynamic load impact test is divided into two parts: the conventional dynamic compression test and dynamic and static combined loading test. Specific test protocols are shown in Table 1.

## 3. Main Results of the Conventional Dynamic Uniaxial Compression Test

### 3.1. Dynamic Stress–Strain Curves

**Figure 2:**
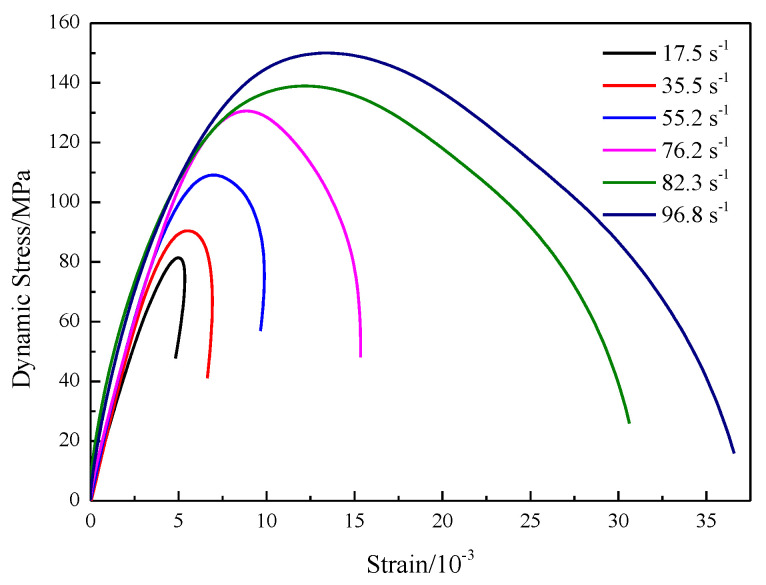
Stress–strain response of medium sandstone at various strain rates.

In the conventional dynamic uniaxial compression tests, three specimens were tested for each strain rate. In the case of each test, with the dynamic stresses in equilibrium, the average dynamic stresses and stress–strain profiles of the specimens were obtained using Equation (2). Without loss of generality, the typical dynamic stress–strain curves for the specimens are plotted in Figure 2.

The dynamic stress–strain curve partially shows linear deformation compared to the static stress–strain curve, implying that crack closure may not occur because the pre-existing microcracks are not closed in a timely manner under a higher loading rate. This result agrees well with the experimental results obtained by other researchers [33]. After the pseudo linear period, the dynamic stress–strain curve is slightly concave upward until it reaches the peak strength, indicating that inelastic deformation is generated in the specimen due to crack initiation and growth. Once the peak strength was attained, the loading capacity of the specimens decreased, and, finally, the specimens failed.

As shown in Figure 2, data suggest that the variation in the dynamic stress–strain curves shows strain-rate dependence. Dynamic stress–strain curves were divided into two groups: Class I (at low strain rates of 17.5 to 76.2 s^−1^) and Class II (at high strain rates of 82.3 to 96.8 s^−1^). Class I has the characteristic that the slope of the stress–strain curve is positive at the postpeak stage. When the dynamic strength increased to the peak strength, lateral splitting failure occurred, which was attributed to tension stress induced by the Poisson effect.

The fragment shape was intact and single-fractured (e.g., the strain rate was 17.5 s^−1^). In contrast to Class I, Class II had a negative slope, and substantial residual strain occurred at the postpeak stage and induced pulverized fragments with all loss of cohesion. At low strain rates, a small proportion of input work by striker impact was consumed for dynamic fragmentation. Due to a few longitudinal fragments, the energy dissipation was limited. As a result, the remaining input work was converted to elastic energy release in the form of recovery of strain.

These elastic energy releases drove successive strain recovery; thus, the stress–strain curves behaved as Class I. As the strain rate increased, crack growth and propagation were activated and nucleated into the fracture surface. Then, localized pulverization was generated. To drive successive crack growth and propagation, elastic energy release was limited, and most of the input work was dissipated in the form of inelastic deformation. Finally, the stress–strain curves behaved as Class II at high strain rates.

### 3.2. Dynamic Elastic Modulus

**Figure 3:**
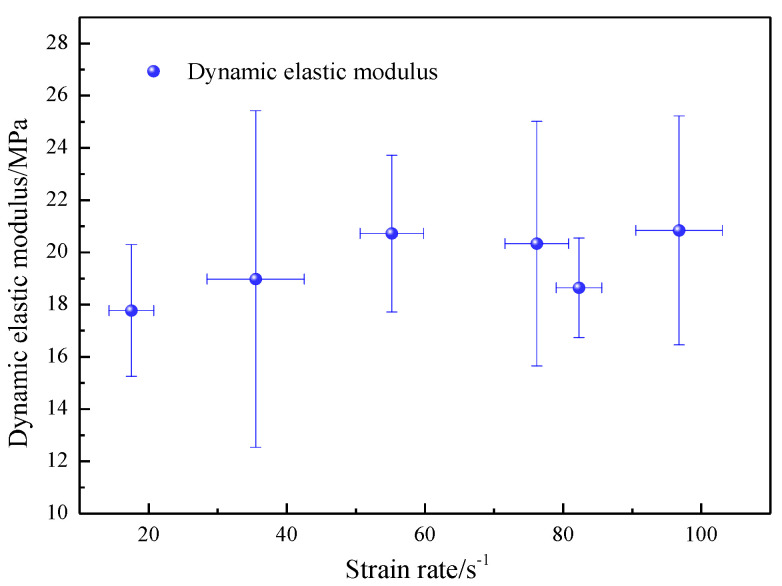
Influence of the strain rate on the dynamic elastic modulus of medium sandstone.

The ISRM-suggested dynamic compression testing methods contain no clear definition for measuring the dynamic modulus of rocks. Therefore, the method recommended by Zhou et al. [31] was used for the determination of the dynamic modulus of elasticity. In the suggested method, a dynamic stress–strain curve is chosen to extract the dynamic modulus of elasticity. By calculation, the dynamic elastic modulus is shown in Figure 3.

**Figure 4:**
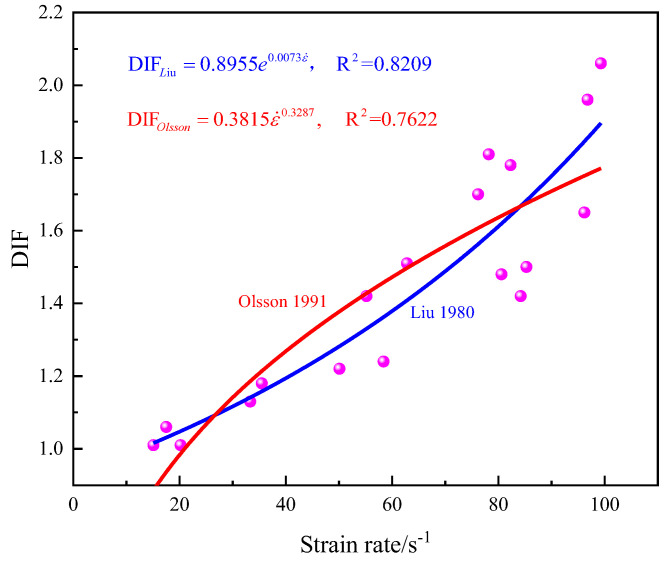
DIF for medium sandstone at different strain rates.

Figure 3 shows the influence of the strain rate on the dynamic elastic modulus. Figure 4 indicates that the dynamic elastic moduli remained constant and were scattered when the strain rate increased from 17.5 to 96.8 s^−1^. Zwiessler et al. [34] suggested that, with an increasing strain rate, the dynamic elastic modulus was scattered. Goldsmith et al. [35] observed that the dynamic elastic modulus increased with the strain rate. This article concluded that there was no agreement about the relationship between the dynamic elastic modulus and strain rate. Under a range of 17.5 to 96.8 s^−1^, the dynamic elastic modulus of the sandstone was independent of the strain rate.

### 3.3. Rate-Dependent Dynamic Uniaxial Compressive Strength

Previous studies have shown that the loading rate influences the dynamic uniaxial compressive strength [36]. In this section, the dynamic increase factor (DIF) is the ratio between the dynamic strength and the static strength, it can be used to evaluate the rate dependency for rock materials. Figure 4 shows the dynamic increasing factor with changes in the strain rate.

An examination of the DIF indicates a series of characteristic patterns as shown in Figure 4. At a low strain rate, the DIF increased slightly linearly, and its rate was slow. Once the strain rate exceeded the critical strain rate (55.2 s^−1^), the dynamic increasing factor increased sharply, which indicated that the dynamic uniaxial compressive strength was obviously affected by the strain rate. At strain rates of 17.5 and 55.2 s^−1^, the dynamic increasing factors were approximately 1.06 and 1.18, respectively; specifically, the dynamic uniaxial compressive strength was 81.20 MPa. When the strain rate was 96.8 s^−1^, its dynamic increasing factor was clearly higher than the dynamic increasing factor of the former two because of inhibiting crack development.

To describe the effect of the strain rate on the enhancement of rock strength, in the case of this study, reference use the extended model form of $\sigma_{dyn}=\sigma_{sta}e^{c\varepsilon }$ Liu [37], $\sigma_{dyn}=\sigma_{sta}a\varepsilon^{n}$ and Olsson [38] to fit the experimental results. The results show that existing empirical laws could be used to describe the rate-dependent strength, but the Liu model for fully describing dynamic behavior is good. The regressed result presented as $DIF=0.8955e^{0.0073\varepsilon }$ has the best regression value R^2^ = 0.8209. Therefore, this article suggest that this result could be used to describe the change in DIF for medium sandstone at different strain rates.

## 4. Coupled Static–Dynamic Compression Test

### 4.1. Dynamic Stress–Strain Curves

**Figure 5:**
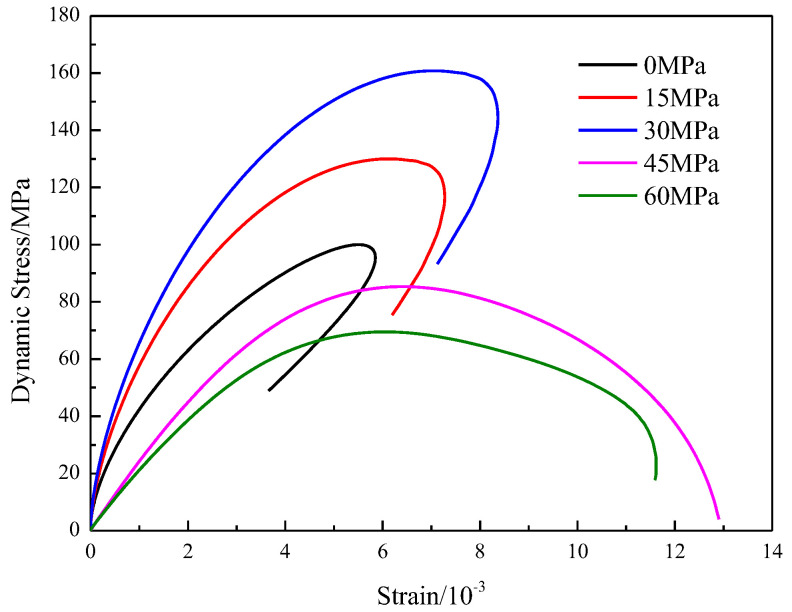
Dynamic stress–strain curves of medium sandstone at different prestatic stresses.

Figure 5 shows the results from coupled static–dynamic compression experiments. For comparison, the stress–strain curve from conventional uniaxial compression tests is plotted with the strain rate.

Figure 5 shows that the dynamic stress–strain curves for all tests can be divided into three stages, i.e., the linear elastic deformation stage; the crack initiation, development and growth stage; and the postpeak stage. The crack closure stage that appeared in the static stress–strain curve was also not observed in the dynamic stress–strain curves. Two explanations for this missing data are possible. Either the microcrack closure process was completed under the initial prestatic stress, or the pre-existing microcrack did not generate timely closure because of the very short impulse duration in the dynamic tests.

In Figure 5, when the axial static load is 0, 15 or 30 MPa, the stress–strain curve appears rebound phenomenon, the lower static load does not make the specimen reach the plastic damage stage, and the damage is dominated by dynamic load at this time; when the static load is 45 or 60 MPa, the stress–strain curve is “open type”, the higher static load makes the specimen enter the plastic stage faster, and the damage of the specimen is dominated by static load and induced by dynamic load at this time, which is consistent with the existing literature.

Interestingly, for our ‘‘coupled static–dynamic compression’’ experiments, Class I and Class II existed in the postpeak stage. Figure 5 also indicates that the transition from Class I to Class II is governed by the applied prestatic stress. When the specimens were subjected to prestresses of 0, 15 and 30 MPa, the stress–strain curve exhibited Class I mechanical behavior. However, for specimens under 45 and 60 MPa, prestatic Class II stress was observed. This result indicates that the higher the initial prestatic stress is, the more energy consumption there is in relation to rock fragmentation.

### 4.2. Dynamic Modulus and Strength

**Figure 6:**
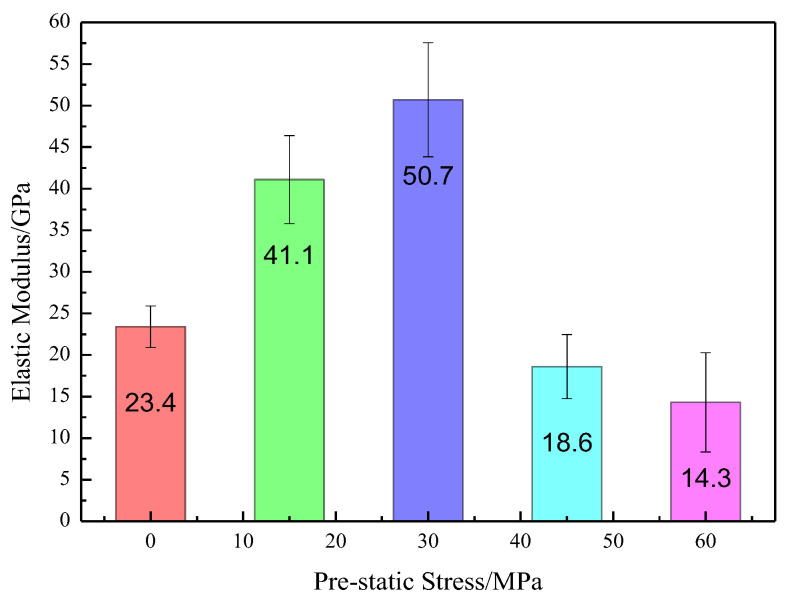
Influence of prestatic stress on the dynamic elastic modulus of medium sandstone.

As observed in Figure 5, the stress–strain characteristics seem similar before failure. However, different deformation behavior after failure is observed. Figure 6 shows the influence of the initial prestatic stress on the dynamic elastic modulus.

As shown in Figure 6, with increasing initial prestatic stress, the dynamic modulus of elasticity first increases non-linearly and then decreases. When the initial prestatic stress was 30 MPa, the dynamic elastic modulus reached the maximum value of 50.7 GPa, which is more than twice the value of the specimen without initial prestatic stress. However, when the initial prestatic stress reached 60 MPa, the dynamic elastic modulus was approximately 71.8% smaller than the maximum elastic modulus.

**Figure 7:**
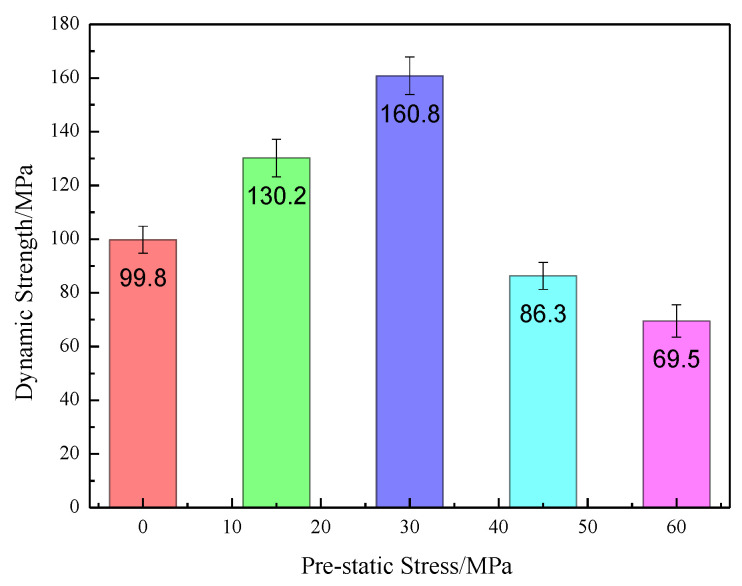
Influence of prestatic stress on the dynamic strength of medium sandstone.

Enhancement of the static prestress on the specimen during dynamic tests can also lead to specimen failure and exhibit mechanical behavior different from the behaviors of conventional dynamic uniaxial compression tests. The dynamic strength under various initial prestatic stresses is shown in Figure 7. The results in Figure 7 show that the initial prestatic stress affects the dynamic strength of the medium sandstone samples.

When the initial prestatic stress was 0–40% of the UCS for the medium sandstone samples, the dynamic strength was enhanced up to 160.8 MPa, more than 1.5 times the dynamic strength of the specimen without prestress. However, at a higher initial prestatic stress of 40–80% of the UCS, a weakening effect was observed. In Figure 7, the dynamic strength decreases to 69.5 MPa. This result coincides with previous results for granite and marble samples in SHPB tests [33].

**Figure 8:**
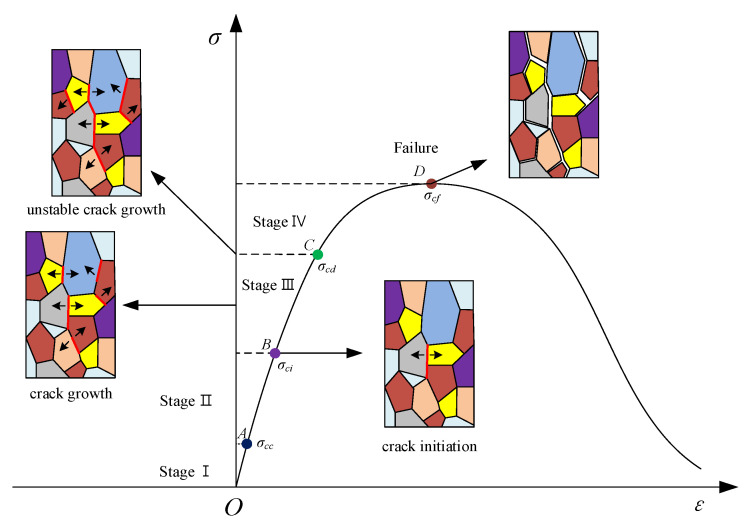
Typical stress–strain response recorded in a uniaxial compressive test.

This mechanism transition from an enhancing effect to a weakening effect of rock dynamic stiffness and strength under coupled static–dynamic compression tests can be explored based on rock damage. In general, the prepeak stress–strain curve of brittle rock samples subjected to uniaxial compression can be divided into four stages: (I) crack closure stage; (II) elastic deformation stage; (III) stable crack growth stage; and (IV) unstable crack growth stage [39]. Experimental evidence demonstrated that the transition from one stage to the next was characterized by the following stress thresholds (Figure 8): crack closure stress threshold σ_cc_, crack initiation threshold σ_ci_ and crack damage thresholds σ_cd_.

When the initial prestress fell in the range of 0–40% of the UCS, specifically, below σci, the closure of pre-existing microcracks or pores occurred, which weakened the initial damage of the rock specimen owing to the reduction in the initial microcrack density. As a result, under dynamic loads, the stiffness and strength of rock materials were enhanced. However, the enhancing effect was limited when the initial prestatic stress exceeded 40% of the UCS, the activation and growth of microcracks in the specimen were generated, and then the initial damage of the rock specimen was pronounced, causing a reduction in rock stiffness and strength under the same dynamic loads.

## 5. Discussion

### 5.1. The Effect of Strain Rate on Rock Dynamic Failure

**Figure 9:**
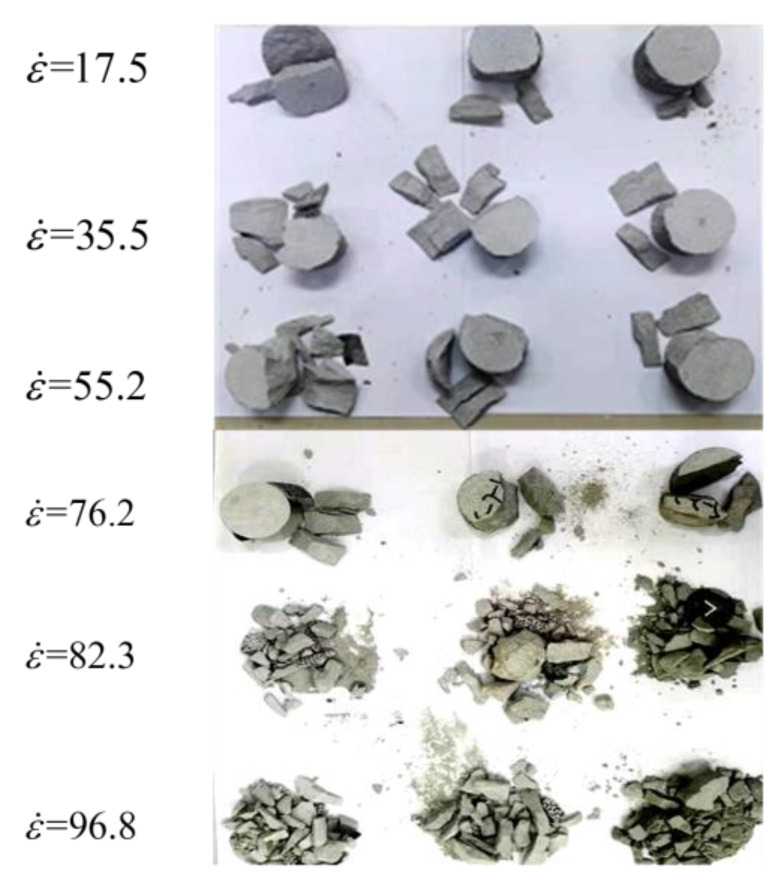
The failure modes of medium sandstone specimens from conventional dynamic uniaxial compression experiments.

As described in Section 3.1, two mechanical classes (Class I and Class II) can be observed in conventional dynamic uniaxial compression tests, which, in turn, results in rate dependency of the failure mode. Figure 9 displays the failure modes of medium sandstone specimens from our conventional dynamic uniaxial compression experiments.

Figure 9 shows that, with the increase in strain rate from 17.5 to 96.8 s^−1^, the degree of failure increased rapidly. At $\dot{\varepsilon }$ = 17.5 s^−1^, our medium sandstone specimens all demonstrated a single or double fractured surface near the free boundaries. The extension of the crack along the axial direction of the medium sandstone specimens led to the final lateral splitting mode. Our postmortem specimens also indicated that the fracture surface was very smooth and flat and that there was no obvious friction trace, which was the result of tension-crack propagation along the loading direction. From the above, tension failure occurred more readily at a lower strain rate. At a strain rate of 76.2 s^−1^, the sample was split into fragments in the form of pieces parallel to the loading direction.

The failure mode was also characterized by lateral splitting. However, a dynamic load in the range of $\dot{\varepsilon }$ = 17.5 to 76.2 s^−1^ did result in the main fracture but led to the extension of other fractures parallel to the direction of loading. Hence, Class I failure resulted from tensile stress as shown in Figure 9. However, at a strain rate of 96.8 s^−1^, samples subjected to dynamic loading were pulverized because of Class II mechanical behavior, and a large number of visible pulverizations were observed, implying the formation of a fracture network distributed homogenously in the sample. In association with previously described results, the rate dependency of the failure mode was characterized by the macroscopic end states: single break, fragmentation or crushing.

**Figure 10:**
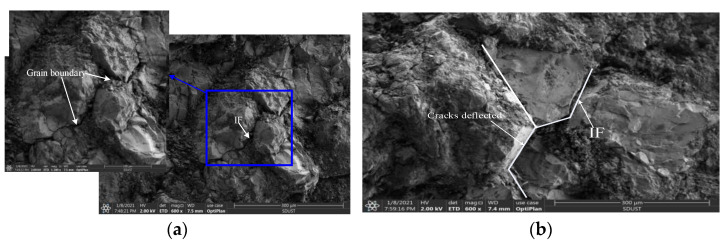

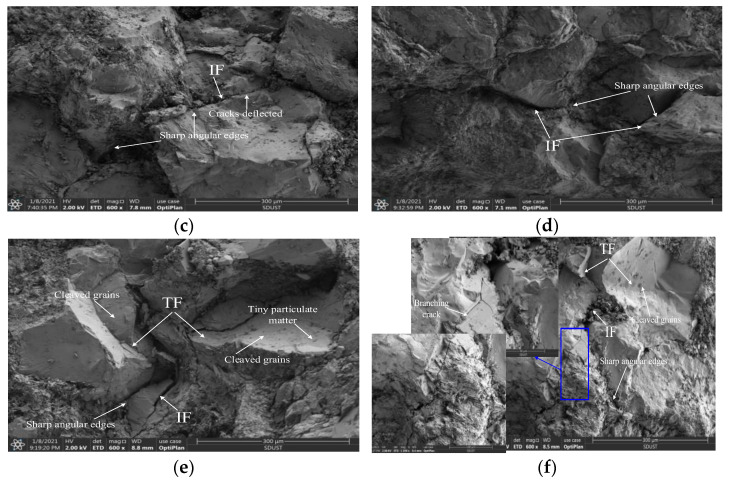
SEM images of typical medium sandstone fractures at various strain rates: (**a**) 17.5 s^−1^; (**b**) 35.5 s^−1^; (**c**) 55.2 s^−1^; (**d**) 76.2 s^−1^; (**e**) 82.3 s^−1^; and (**f**) 96.8 s^−1^.

The transition of dynamic failure from Class I failure to Class II failure can be collected after the sample’s failure status; thus, the micro-characteristics of crack fracturing transition capture seem to be unavailable. Furthermore, specimens with a typical fracture surface from our tests were selected, and the results of the SEM images are shown in Figure 10. The SEM examination of micrographs shows that, from Figure 10a–d, in Class I tests, most cracks deflected along the grain boundaries, resulting in a sharp angular edge. This observation indicated that IF was the predominant form of cracking on the fracture surface of specimens that fractured with Class I mechanical behavior.

A different phenomenon can be observed for the SEM images with the same magnification in Class II tests. Figure 10e shows that the fractures seem flatter, and cleaved grains are apparent. Much tiny particulate matter is left behind by shear friction. In addition, typical IF was also found in Figure 10e. TFs were found in Figure 10f, which is similar to Figure 10d. Furthermore, a typical branching crack that may be associated with the formation of a fracture network appears to be found on the sections of the fractured specimens. Therefore, Class II failure was dominated by combined IF and TF processes in the microscopic view.

As stated above, microfission patterns related to the rate can be divided into two main forms, and, in turn, this transition results in two macrofailure patterns as shown in Figure 10. On a smaller scale, some microcracks were generated first because of reflected tensile waves between grain boundaries. As the stress wave travels through the specimen, the activated microcracks start to propagate and then meet the mineral grains. In terms of fracture mechanics, the fracture toughness of grain boundaries is generally larger than the resistance of the grain material to crack propagation. Therefore, secondary cracking happens at the tip of the crystal line, and the propagating crack deflects into the interface, resulting in a rough fracture surface (denoted as IF).

Due to the subsequent energy input, these secondary cracks propagate and nucleate into the fracture surface along the loading direction and cause Class I failure. Upon increasing the loading rate, the energy release rate at the crack tip is sufficiently large to overcome the resistance of the grain to crack propagation. Then, the propagating crack directly penetrated to the target grain (denoted as a TF). When the external energy is sufficiently high, adjacent secondary cracks nucleate into crack clustering (this type of failure aims to consume a large amount of energy in a short impulse duration), which eventually leads to the macropulverization phenomenon.

### 5.2. The Effect of Initial Prestatic Stress on Rock Dynamic Failure

**Figure 11:**
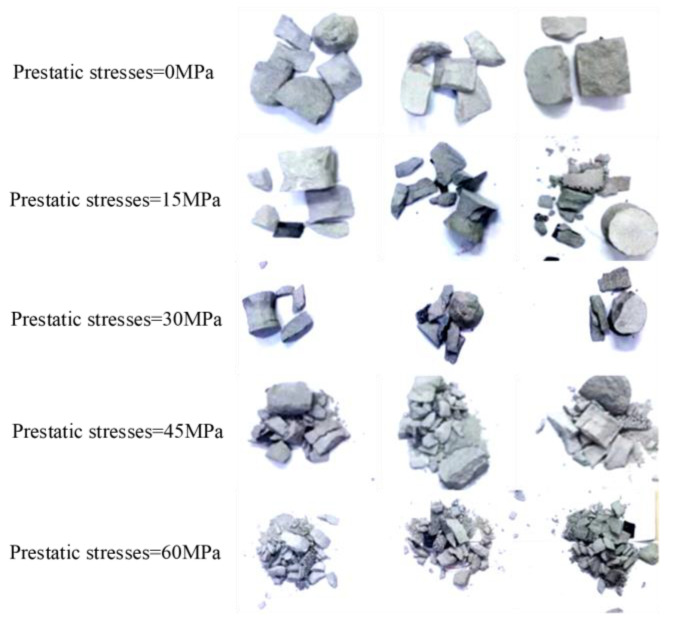
Typical failure modes of sandstone specimens subjected to coupled static–dynamic compression.

**Figure 12:**
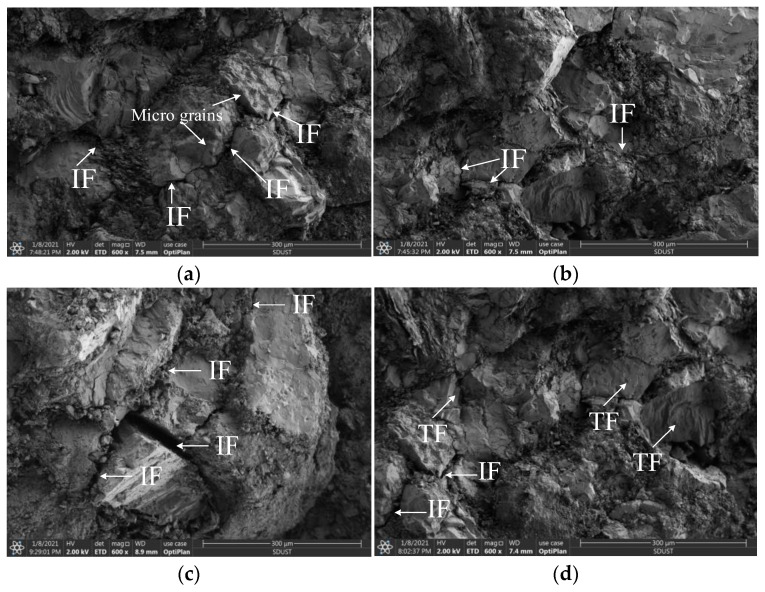

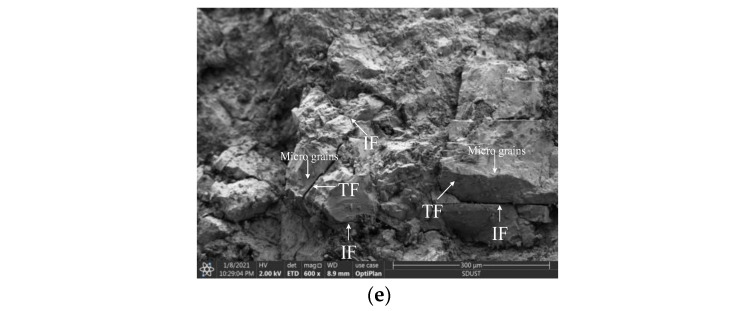
SEM images of typical medium sandstone fractures at various prestatic stresses: (**a**) 0 MPa; (**b**) 15 MPa; (**c**) 30 MPa; (**d**) 45 MPa; and (**e**) 60 MPa.

Figure 11 shows the typical failure modes of sandstone specimens subjected to coupled static–dynamic compression. Figure 12 shows that, at initial prestatic stresses of 0, 15 and 30 MPa, the sandstone material exhibits a typical pattern of transverse splitting (Class I damage). In the microscopic view, some microcracks initiated and kinked out to propagate along the grain boundaries, leaving most of the grains intact. This behavior indicates that the fracture mode of medium sandstone specimens subjected to initial prestatic stress of 0–30 MPa was mostly an intergranular crack pattern that is characterized by tension failure. At initial prestatic stresses of 45 and 60 MPa, the medium sandstone specimens were nearly pulverized and impacted into small pieces (Class II failure).

On a smaller scale, in addition to the IF, many smaller, isolated TFs are recognized in the SEM image, representing apparent shear failure. Upon increasing the initial prestatic stress, the macroscopic end state of the specimens was observed to transition from fragmentation to pulverization. Similar to the conventional dynamic uniaxial compression tests, the different microscopic failure modes were the root cause of the transition in the stress-dependent macroscopic failure of sandstone specimens.

The microcracking process is closely related to the macroscopic failure of rocks subjected to dynamic loading. A global descriptiveness of the microcracking process can enhance the understanding of stress-dependent damage in rocks from a microscopic perspective. In association with the described results, the stress-dependent microcracking process was categorized into two forms.

This article can conclude that: (a) In Class I loading tests (where the prestatic stress was less than σ_ci_), inherent micro-defects, such as microcracks and voids, closed first. As the fracture toughness of cross-grain contact is generally greater than the fracture toughness of intergranular contact, most microcracks were randomly generated at the grain interfaces, and these activated microcracks deflected along the grain in response to stress impulses, which resulted in secondary cracks. Once the external energy was sufficiently large to drive the propagation of secondary cracks, secondary cracks coalesced to the main fracture surface and then resulted in the ultimate Class I failure. This is similar to the study in the literature [40].

(b) Under Class II loading test conditions, intergranular cracks were generated because of the prestatic stress concentration. The progressive propagation of these generated cracks was determined by the subsequent energy input. As the external energy was sufficiently high, propagated microcracks deflected directly into the interface or penetrated the grain. These cracks usually nucleated into crack clusters and led directly to macroscopic comminution phenomena.

## 6. Conclusions

In this study, an SHPB experimental system was used to perform dynamic loading tests on typical medium sandstone samples, and SEM was used to analyze the microfracture characteristics of broken samples. The following conclusions were reached:

In the CDUC tests, when the strain rate increased from 17.5 to 96.8 s^−1^, the dynamic uniaxial compressive strength increased, and the dynamic elastic modulus was scattered. However, based on the results obtained by CDUC, the rate dependency of the failure mode was characterized by the macroscopic end states. It is known from these experiments that Class I samples were subject to strain rates ranging from 17.5 to 76.2 s^−1^, in which some samples were intact or slightly fractured fragments. Class (II) samples were subject to a higher strain rate (82.3 s^−1^ ≤ $\dot{\varepsilon }$ ≤ 96.8 s^−1^). Some of these samples were sufficiently disrupted to become crushed.

In the CSDL tests, the dynamic elastic modulus and dynamic strength first increased nonlinearly and then decreased when the initial prestatic stress increased from 0 to 60 MPa (approximately 78% of UCS). Two classical mechanical types were also observed as initial prestatic stresses from 0 to 60 MPa. Class I only existed when the prestatic stress was less than the crack initiation threshold, in which the failure state was characterized by single fracturing or several fragments parallel to the direction of stress wave propagation. In contrast to Class I, Class II was subject to a higher prestatic stress with microcrack activation.

The dominant microcrack transition was from intergranular to transgranular cracks, resulting in different macroscopic mechanical types. Based on the SEM analysis of medium sandstone fractures observed from either CDUC loading or CSDL loading, intergranular fracture occurred in Class I tests. In contrast, Class II behavior was the result of the combined IF and TF.

## Figures and Tables

**Table 1:** Test plan.

Load Type	Variable	Gradient
General dynamic compression	strain rate	From low to high
Dynamic and static combined loading	Axial stress	0 MPa 15 MPa 30 MPa 45 MPa 60 MPa

## Data Availability

Data available upon reasonable request to author(s).
